# Anti-inflammatory effects of CBD in human microglial cell line infected with HIV-1

**DOI:** 10.1038/s41598-023-32927-4

**Published:** 2023-05-05

**Authors:** Adriana Yndart Arias, Nagesh Kolishetti, Arti Vashist, Lakshmana Madepalli, Lorgeleys Llaguno, Madhavan Nair

**Affiliations:** 1 Department of Immunology and Nano-Medicine, Herbert Wertheim College of Medicine, Florida International University, Miami, FL 33199 USA; 2 Institute of Neuroimmune Pharmacology, Herbert Wertheim College of Medicine, Florida International University, Miami, FL 33199 USA

**Keywords:** Inflammasome, Neuroimmunology, Neuroscience, Immunology, Infectious diseases, Inflammation, Neuroimmunology

## Abstract

Human immunodeficiency virus (HIV) infection is associated with a chronic inflammatory stage and continuous activation of inflammasome pathway. We studied the anti-inflammatory effects of the compound cannabidiol (CBD) in comparison with Δ (9)-tetrahydrocannabinol [Δ(9)-THC] in human microglial cells (HC69.5) infected with HIV. Our results showed that CBD reduced the production of various inflammatory cytokines and chemokines such as MIF, SERPIN E1, IL-6, IL-8, GM-CSF, MCP-1, CXCL1, CXCL10, and IL-1 β compared to Δ(9)-THC treatment. In addition, CBD led to the deactivation of caspase 1, reduced NLRP3 gene expression which play a crucial role in the inflammasome cascade. Furthermore, CBD significantly reduced the expression of HIV. Our study demonstrated that CBD has anti-inflammatory properties and exhibits significant therapeutic potential against HIV-1 infections and neuroinflammation.

## Introduction

Cannabis is one of the most commonly used drugs worldwide, as reported by the United Nations Office on Drugs and Crime in 2020. It contains the main psychoactive component Δ-9-tetrahydrocannabinol (Δ(9)-THC)^1^ and the non-intoxicating compound cannabidiol (CBD). Δ(9)-THC and CBD are phytocannabinoids that act on the endocannabinoid system^1^. Δ (9)-THC has a high binding affinity for cannabinoid receptors 1 and 2 (CB1R = 25.1; CB2R = 35.2 nM) in the nanomolar (nM) range, while CBD has 80 fold lower binding affinity to these receptors^2^. Δ(9)-THC is considered a potent psychotropic element of cannabis, causing euphoria when used. Its effects are attributed to the interaction with the endocannabinoid system, which includes cannabinoid receptors (CB1 and CB2), endocannabinoids (anandamide, 2 arachidonoylglycerol), and enzymes involved in their breakdown. On the contrary, CBD is considered non-psychoactive due to its biological effects on the central nervous system (CNS) without producing euphoria. The medicinal cannabis contain a wide variety of cannabis compounds, in major quantity the Δ(9)-THC, which generates the reported adverse events. However, preparations containing a high percentage of CBD exerts antioxidant, antidiabetic, anticancer, anxiolytic, anticonvulsive, antipsychotic, antiepileptic, and anti-inflammatory properties^3^. The molecular action behind these beneficial effects may be due to the inhibition of the reuptake and hydrolysis of anandamide. Additionally, CBD has been shown to reduce drug reward and addiction in part by the negative allosteric modulation of CB1R, its partial or invert agonist action on CB2R, its agonist action on transient receptor potential vanilloid 1 (TRPV1), and serotonin 5-HT1A receptor^4–6^.

Microglia are resident macrophages cells in CNS and account for 5–12% of total cells in the CNS. They actively defend the brain against infections and inflammation, eliminating debris, recruiting immune cells, promoting neurogenesis. Microglial cells quickly respond to pathological stimuli and activate the initiation of responses that promote neuronal protection or pathogenesis. Moreover, it is known that infections can trigger inflammation and microglial activation thus disrupts brain homeostasis^7–9^.

People living with HIV consume 2–3 times more cannabis than general population. About 14–56% of them use this drug mainly to manage nausea, musculoskeletal and neuropathic pain, sleep disorders, anxiety, and depression^10^.

Patients diagnosed with HIV exhibit high levels of chronic systemic inflammation, which may drive the development of comorbidities such as cardiovascular diseases, diabetes, and neurocognitive disorders. Although, the HIV-associated neurocognitive disorder (HAND) is not fully understood, the neurocognitive disorders in these patients have been associated with chronic neuroinflammation, leading to neurodegeneration, increased microglia and astrocyte activation, dysfunction, damage, and ultimately death of neurons^11,12^.

Although the entrance of pathogens and specific substances is limited in the brain by the blood–brain barrier (BBB), HIV enters through the infected peripheral blood monocytes/macrophages that can cross the BBB. The infected immune cells inside the brain then propagate the infection to the resident macrophages (microglia) and astrocytes. Moreover, the use of controlled substances such as cocaine, methamphetamine in HIV patients exacerbate neuroinflammation, HIV infection, and neuronal damage^13^. In addition, the fact that many of the antiretroviral drugs are impermeable to the BBB^11,13^ made the brain a repository for HIV, and leads to cognitive decline in patients. Thus, finding alternative treatments that can reduce the inflammatory process in HIV patients is crucial.

Based on previous evidence anti-inflammatory properties and the absence of euphoria and addiction, we conducted a systematic study to evaluate the mechanistic effects of CBD on microglial cells, including inflammasome activation and inflammatory cytokines production, and compared to the effects of Δ(9)-THC treatment.

## Results

### The XTT assay and cytotoxicity

**Figure 1:**
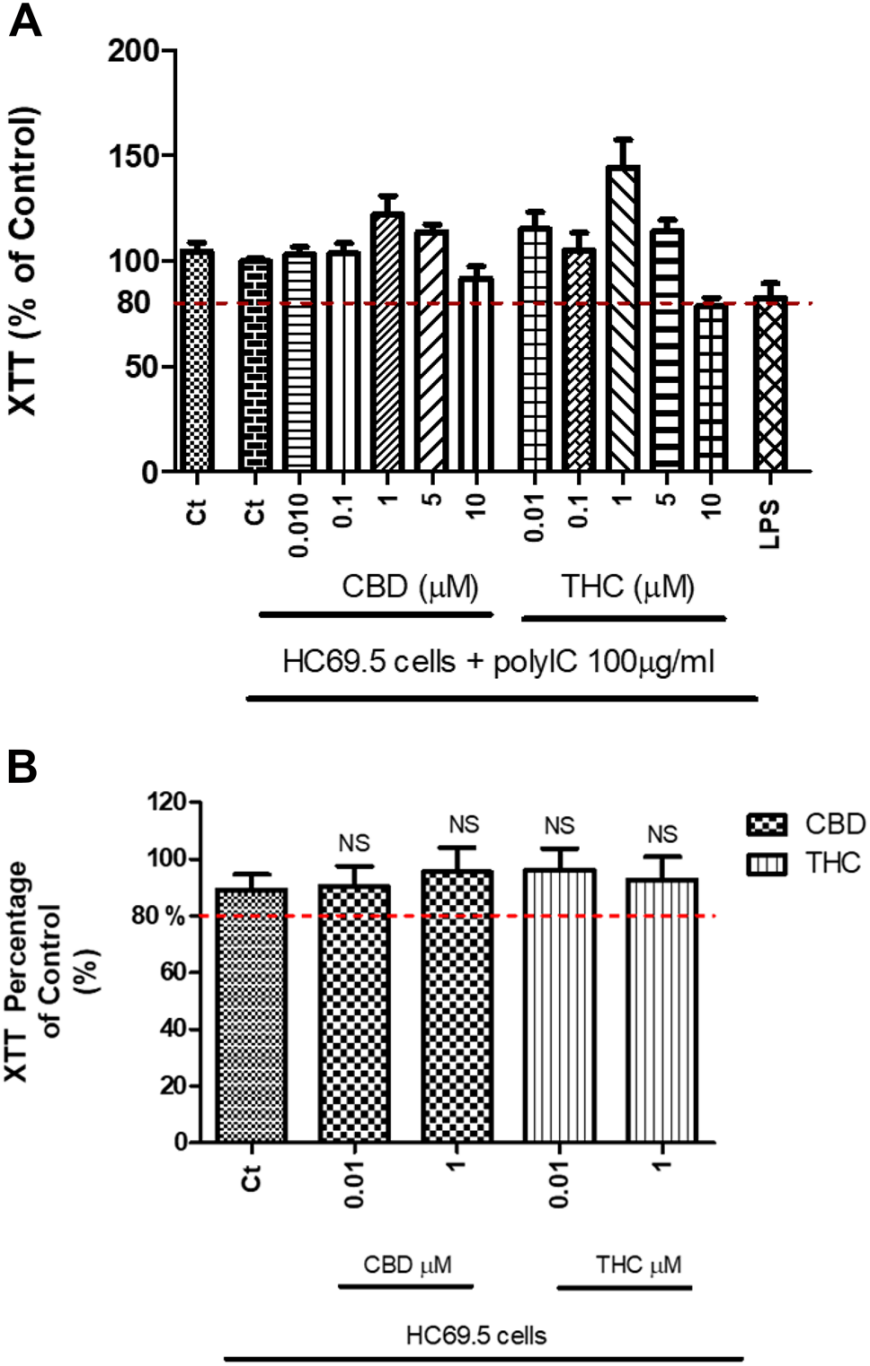
(**A**) HC69.5 cells were treated with 100 µg/ml of poly IC and different concentrations of CBD or THC. After 24 h of drug treatment, the XTT-activated reagent was added. Cells treated with LPS were used as positive control. (**B**) Similarly, cells without poly IC were treated with different concentrations of CBD or THC. After 24 h of drug treatments, the XTT-activated reagent was added, and absorbance was measured after 4 h. Results are presented as a percentage of control, untreated HC69.5 cells. GraphPad Prism 5 software was used to plot and conduct statistical analysis. Normality distribution was checked by Kolmogorov–Smirnov test, while ANOVA and Dunns calculated statistical significance as a post-hoc test. Experiments were performed at least three times in replicates.

The HC69.5 (HIV/GFP+) microglia cells were activated with poly IC (100 μg/ml) and treated with different concentrations of CBD and Δ (9)-THC (0.001 μM to 1 μM) for 24 h. Cytotoxicity was measured using XTT assay, and data presented in Fig. 1 did not shown any significant cytotoxicity in any of the test groups when compared with the control cells (Fig. 1).

### HIV reactivation in microglial cells

**Figure 2:**
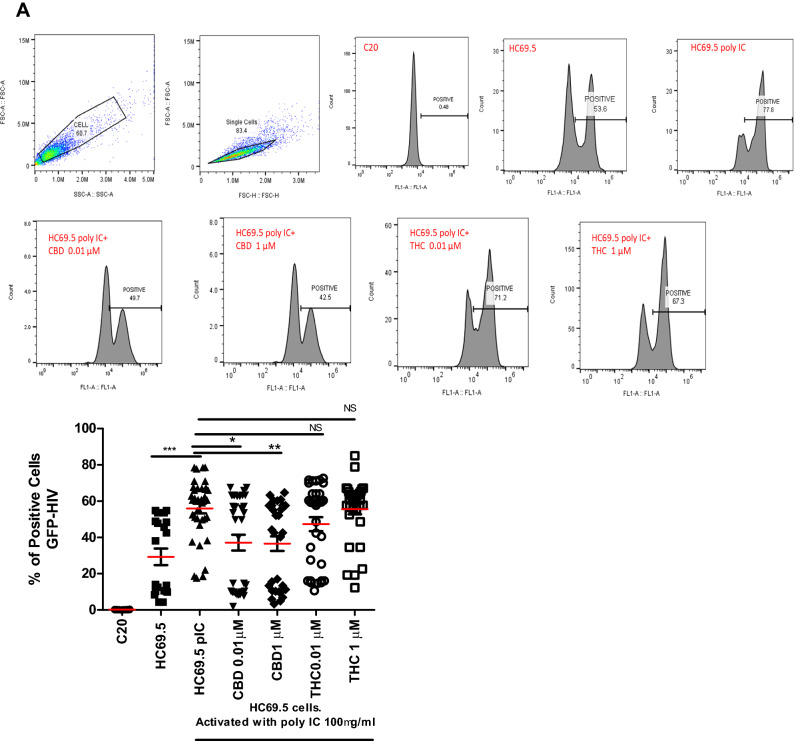

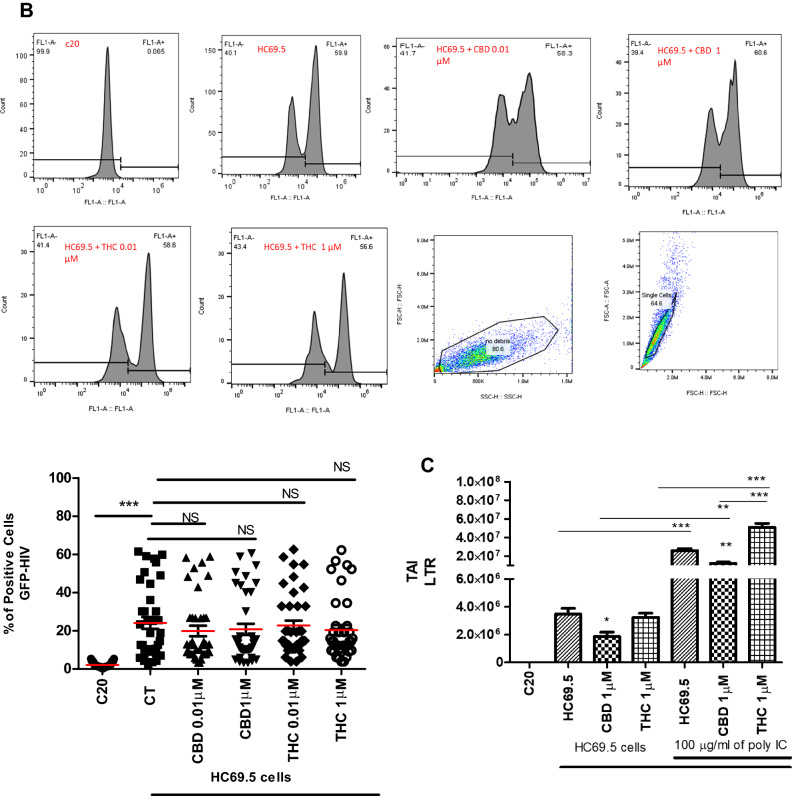
(**A**) HC69.5. HIV (GFP+) cells were treated with 100 μg/ml poly IC and different concentrations of Δ (9)-THC or CBD (0.01 and 1 μM). Cells were incubated overnight and were fixed using the Fixation and Permeabilization Solution Kit (Cat. No. 554715). Flow cytometry analysis was performed to detect the HIV infection by expressing GFP-positive cells. Immortalized human microglia cells (C20) (HIV-; GFP-) were used as untreated and uninfected control. Results were expressed as percentage of positive cells. Experiments were performed at least three times in replicates. GraphPad Prism 5 software was used to graph and conduct statistical analysis. The normality distribution was evaluated by Kolmogorov–Smirnov test, while statistical significance (p ≤ 0.05) was calculated by ANOVA and Dunns as a post-hoc test. (**B**) HC69.5. HIV (GFP +) cells were treated with different concentrations of Δ (9)-THC or CBD (0.01 and 1 μM). Cells were incubated overnight and were fixed using the Fixation and Permeabilization Solution Kit (Cat. No. 554715). Flow cytometry analysis was performed to detect the HIV infection by expressing GFP-positive cells. Immortalized human microglia cells (C20) (HIV−; GFP−) were used as untreated and uninfected control. Results were expressed as number of positive cells. Experiments were performed at least three times in replicates. GraphPad Prism 5 software was used to graph and conduct statistical analysis. The normality distribution was evaluated by Kolmogorov–Smirnov test, while statistical significance (p ≤ 0.05) was calculated by ANOVA and Dunns as a post-hoc test. (**C**) LTR gene expression on HC69.5. HIV (GFP+) cells was assessed by RT PCR. H69.5 cells treated with/without 100 μg/ml poly IC, 1 μM concentration of CBD or Δ (9)-THC as figure is showing. After 24 h of treatments, RNAs were extracted, and reverse transcribed followed by real time PCR for LTR gene (HIV LTR Pa03453409_s1; Thermofisher Scientific). GAPDH (Hs99999905_m1) was used as a housekeeping gene. Data represent the means ± standard error of three independent experiments. Graph represents the transcript accumulation index respect to negative control C20. Data were analyzed using GraphPad Prism software. The normality distribution was evaluated by Kolmogorov–Smirnov test, while statistical significance was calculated by ANOVA and Dunns Multiple Comparison Test as a post-hoc test. Differences were considered significant at p ≤ 0.05.

Flow cytometery was used to detect the expression of HIV/GFP+ cells. After cells were exposed to poly IC for 24 h to induce HIV replication, a significant increase in positive cells was detected (Fig. 2A). Interestingly, adding CBD or Δ(9)-THC to activated HIV cells resulted in a significant decrease in positive cells in only CBD treated cultures. However, Δ(9)-THC treatments showed no significant reduction compared to the activated control (Fig. 2A).

Moreover, no significant differences were found in absence of the poly IC after CBD or Δ(9)-THC treatments (Fig. 2B). The LTR gene expression assay was also conducted, which showed an induction of HIV infection when cells were treated with poly IC. This increase in the LTR gene expression was reduced by adding 1 μM of CBD and 1 µM of THC. Moreover, when HIV was not induced by the addition of poly IC, CBD reduced the expression of the long term terminal gene while THC had no significant differences compared to the control (Fig. 2C).

### Cannabinoid receptors in HIV infected microglial cells

**Figure 3:**
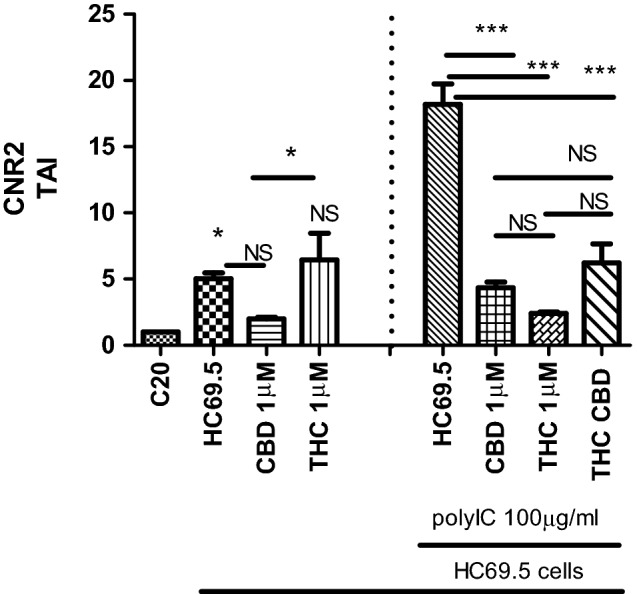
Cannabinoid receptor type 2 gene expression on HC69.5. HIV (GFP+) cells was assessed by RT PCR. H69.5 cells treated with/without 100 μg/ml Poly IC, 1 μM concentration of CBD or /and Δ (9)-THC. After 24 h of treatments, RNAs were extracted, and reverse transcribed followed by real time PCR for CNR2 gene (Hs00275635_m1; Thermofisher Scientific). GAPDH (Hs99999905_m1) was used as a housekeeping gene. Data represent the means ± standard error of three independent experiments. Graph represents the transcript accumulation index respect to negative control C20. Data were analyzed using GraphPad Prism software. The normality distribution was evaluated by Kolmogorov–Smirnov test, while statistical significance was calculated by ANOVA and Tukey Multiple Comparison Test as a post hoc test. Differences were considered significant at p ≤ 0.05.

We investigated the gene expression of CNRs in HC69.5 cells exposed to CBD and/or THC with and without the addition of the poly IC (Fig. 3). We observed an upregulation of CNR2 when HIV was activated by adding the poly IC, TLR3 agonist and significant decrease when cannabinoids were added. There were no significant differences between the THC and CBD used alone or in combination.

A different scenery was obtained when we did not treat cells with poly IC and the HIV infection was not upregulated. In this case, the addition of CBD or THC did not reduce the cannabinoid receptors respect to HIV control (HC69.5). However, a CNR2 gene showed a significant dysregulation (Fig. 3).

### Cytokines expression

**Figure 4:**
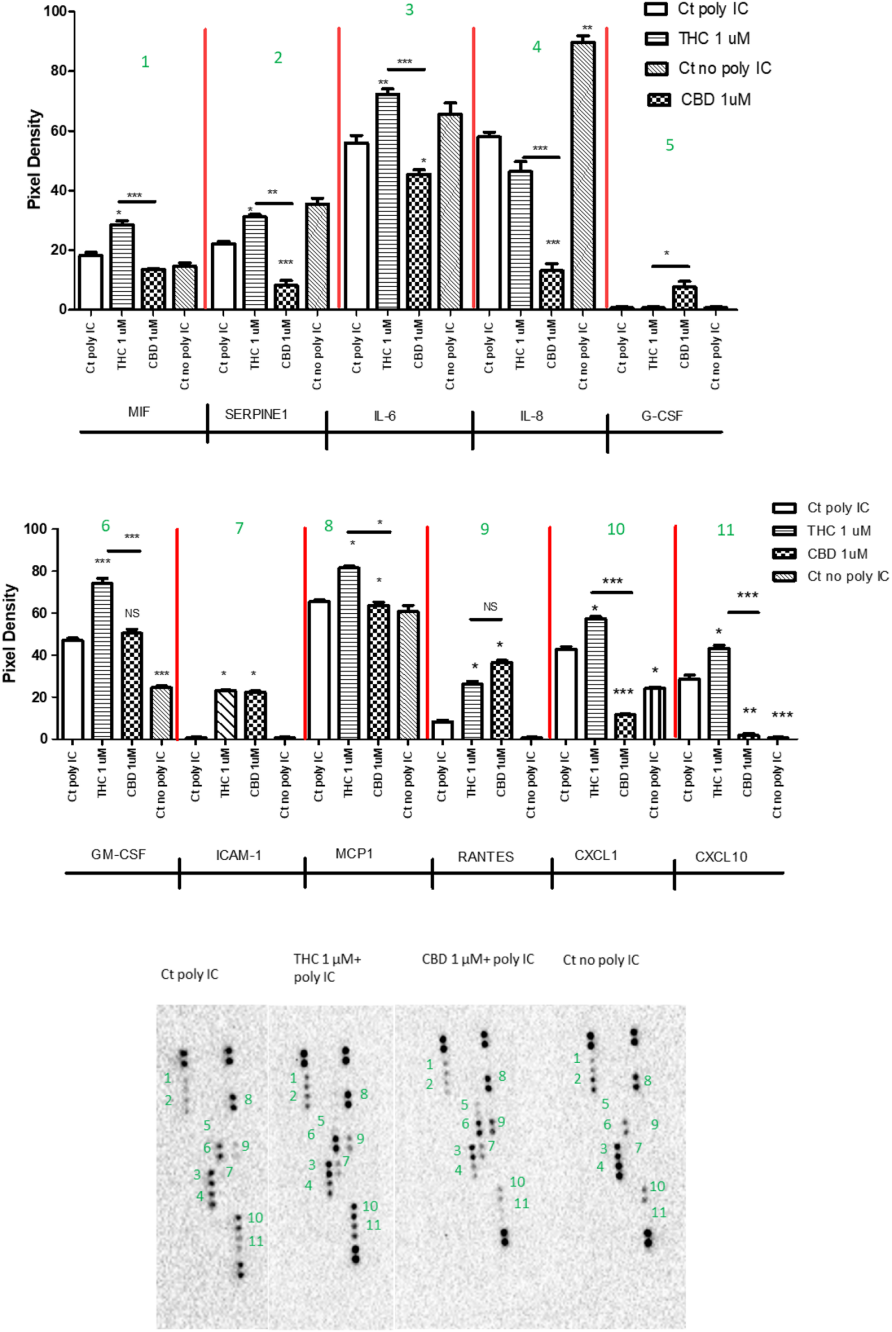
HC69.5 cells were exposed to 1 μM concentration of THC or CBD after HIV activation with 100 μg/ml of poly IC. 48 h after adding the drugs, supernatants were collected and assayed by Proteome Profiler Human Cytokine Array (R&D system: ARY005B) following provider instructions. (Control Ct: control (HC69.5 cells)). GraphPad Prism 5 software was used to graph and conduct statistical analysis. First, normality distribution was checked by the Kolmogorov–Smirnov test for each cytokines. Then, statistical significances (p ≤ 0.05) were calculated by ANOVA followed by Tukey's Multiple Comparison Test as post-hoc test. Each of the dysregulated proteins is numbered in graph and membrane pictures.

We used the Proteome Profiler Human cytokine array to investigate the response of HIV infected microglia cells to CBD and THC treatments. Membrane-based sandwich immunoassay was used to simultaneously demonstrate the differential expression of 36 human cytokines, chemokines, and acute-phase proteins. Supernatants from activated/non-activated cells, treated with 1 μM of CBD or Δ(9)-THC, were mixed with a cocktail of biotinylated detection antibodies, and incubated with capture antibodies specific to target proteins. Membranes were spotted in duplicate for each cytokine or chemokine. Chemiluminescent detection was used to visualize protein expression, and the signal was proportional to the amount of bound molecules. Eleven cytokines were dysregulated (Fig. 4); including MIF, SERPIN E1, IL-6, IL-8, G-CSF, GM-CSF, ICAM-1, MCP-1, RANTES, CXCL1, and CXCL10. 1 μM of CBD reduced the expression of MIF, SERPIN E1, IL-6, IL-8, CXCL1, and CXCL10 that was induced by HIV reactivation in HC69.5 microglia cells, while 1 μM of THC raised the upregulation of many of the cytokines after the reactivation (MIF, SERPIN E1, IL-6, ICAM-1, GM-CSF, MCP-1, CXCL1, and CXCL10). In addition, G-CSF and RANTES were more upregulated by CBD than Δ(9)-THC. However, more studies are needed to elucidate the implications of these dysregulations.

### Caspase 1 dysregulation

**Figure 5:**
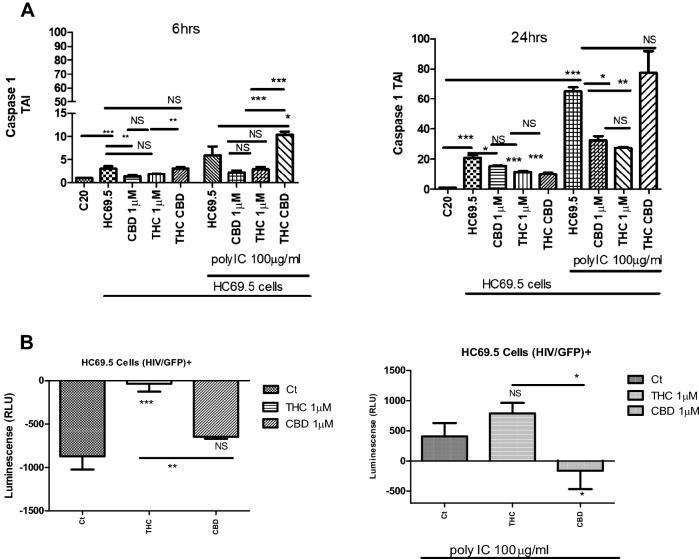
(**A**) Caspase 1 gene expression on HC69.5. HIV (GFP+) cells was assessed by RT PCR. H69.5 cells treated with/without 100 μg/ml Poly IC, 1 μM concentration of CBD or/and Δ (9)-THC. After 6 h and 24 h of treatments, RNAs were extracted, and reverse transcribed followed by real time PCR for Caspase 1 gene (Hs00354836_m1; Thermofisher Scientific). GAPDH (Hs99999905_m1) was used as a housekeeping gene. Data represent the means ± standard error of three independent experiments. Graph represents the transcript accumulation index respect to negative control C20. Data were analyzed using GraphPad Prism software. The normality distribution was evaluated by Kolmogorov–Smirnov test, while statistical significance was calculated by ANOVA and Tukey Multiple Comparison Test as a post hoc test. Differences were considered significant at p ≤ 0.05. (**B**) Caspase 1 Activation. HIV infection in HC69.5. HIV (GFP+) with and without 100 μg/ml poly IC and cells were treated with 1 μM of Δ(9)-THC or CBD. After 24 h. of treatment, Caspase 1 activation was analyzed using Caspase-Glo® 1 Inflammasome Assay kit (Promega; cat: G9951). (Ct = Control). GraphPad Prism 5 software was used to graph and conduct statistical analysis. Normality distribution was checked by the Kolmogorov–Smirnov test. Then, statistical significances (p ≤ 0.05) were calculated by ANOVA followed by Dunn ‘s as a post-hoc test.

The upregulation of caspase-1 mainly activates the inflammasome mediators. We conducted a gene expression assay after 6 and 24 h of treatment (Fig. 5A). Our results showed a significant upregulation of the caspase 1 gene in HC69.5 with and without the addition of poly IC, indicating a certain level of continuous inflammation, inclusively at low levels of HIV infection in microglia cells. When cells were treated with CBD, we observed a significant reduction of the caspase 1 gene after 6 and 24 h of treatments in the absence of polyIC. Likewise, a reduction in the expression of caspase 1 was obtained when the HIV infection was induced by the addition of polyIC at 6 and 24 h, being significant after 24 h. In addition, THC reduced the gene expression of caspase 1, with no significant differences with respect to HC69.5 in the presence or absence of polyIC at 6 h, and significant after 24 h in polyIC added cultures only. The outcomes from the CBD and THC were compared, and the gene expression did not show a significant difference between the two cannabinoids. The combination of THC-CBD showed caspase 1 expression as the HIV only cultures with higher values in the presence of polyIC (Fig. 5A).

The Caspase 1 Glo kit elicits a stable signal proportional to caspase activity, offering a sensitive detection of caspase-1 and inhibiting nonspecific proteasome-mediated cleavage of the substrate. Our results demonstrated that when HIV was not reactivated, CBD and Δ(9)-THC did not enhance the caspase activity. Further, when the cells were treated with poly IC, the caspase 1 was activated and CBD reversed that activation, not in the case of Δ(9)-THC. Thus CBD provided greater protection against caspase 1 activation, a molecule that initiates downstream cleavage to induce an inflammatory response (Fig. 5B).

**Figure 6:**
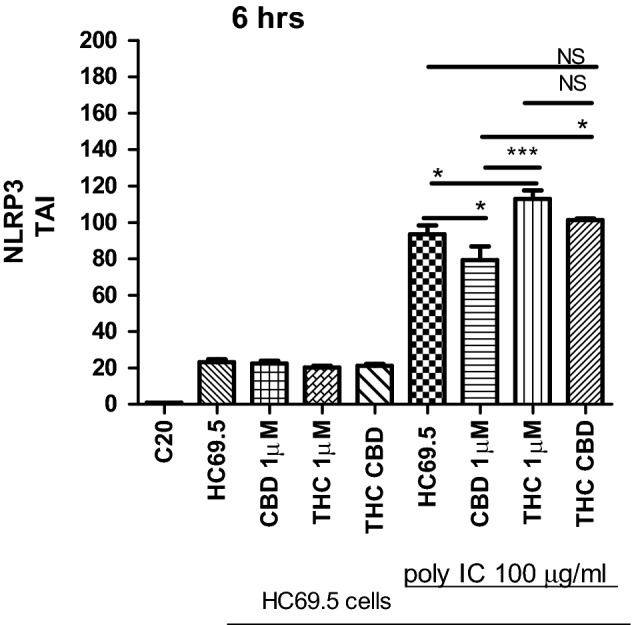
NLRP3 gene expression on HC69.5. HIV (GFP+) cells was assessed by RT PCR. H69.5 cells treated with/without 100 μg/ml Poly IC, 1 μM concentration of CBD or/and Δ (9)-THC. After 6 h of treatments, RNAs were extracted, and reverse transcribed followed by real time PCR for NLRP3 gene (Hs00918082_m1; Thermofisher Scientific). GAPDH (Hs99999905_m1) was used as a housekeeping gene. Data represent the means ± standard error of three independent experiments. Graph represents the transcript accumulation index respect to negative control C20. Data were analyzed using GraphPad Prism software. The normality distribution was evaluated by Kolmogorov–Smirnov test, while statistical significance was calculated by ANOVA and Tukey Multiple Comparison Test as a post hoc test. Differences were considered significant at p ≤ 0.05.

To understand more about how CBD could dysregulate the inflammasome response in HIV infected microglia cells, we examined the gene expression of NLRP3 after 6 h of activation of HIV infection and treatment with cannabinoids (Fig. 6). The NLRP3 inflammasome is a crucial element of inflammasome pathway mediating the activation of caspase-1 and proinflammatory cytokines. Our results show NLRP3 gene is significantly upregulated when the HIV infection is activated, and CBD conferred protection to the rise of NLRP3 expression, which was not observed in the case of THC and cannabinoids combination treatments.

### IL-1ß ELISA

**Figure 7:**
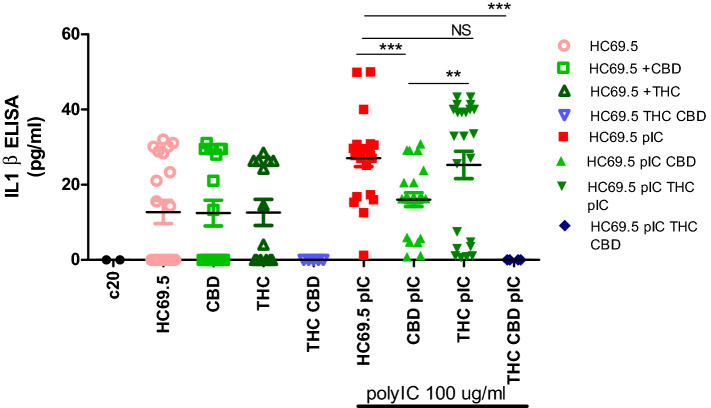
HC69.5. HIV (GFP+) were treated with/without 100 μg/ml poly IC, 1 μM of Δ(9)-THC or CBD. Supernatants were collected after 24 h of treatment and analyzed by ELISA MAX™ Deluxe Set Human IL-1ß (Biolegend; cat# 437004). GraphPad Prism 5 software was used to graph and conduct statistical analysis. Normality distribution was checked by the Kolmogorov–Smirnov test. Then, statistical significances (p ≤ 0.05) were calculated by ANOVA followed by Dunn’s test as post-hoc test.

IL-1ß is a key cytokine in triggering the inflammatory response, and its expression was measured using ELISA. When HIV infection was not induced, neither drug caused a significant expression of IL-1 ß. However, when HIV infection was activated, 1 µM of Δ(9)-THC produced a significant IL-1ß expression after 24 h while CBD suppressed HIV induced IL-1ß expression (Fig. 7). Interestingly, the combination of CBD and THC completely suppressed the IL-1 beta expression. More studies need to be done to understand this reduction.

## Discussion

There is a growing interest in the medical use of cannabis, especially CBD, considering the reported benefits, such as pain reduction, anti-inflammatory properties, antioxidant, antidiabetic, anticancer, anxiolytic, anticonvulsive, antiepileptic, and the absence of psychotropic effects induced by THC^3,14^. The lack of psychotropic effects is one of the most attractive differences from its close derivate THC. However, CBD's regulatory role on inflammasome and HIV infection has not been studied. Previously published studies were focused on the neuroprotective attributes of CBD as well as anti-inflammatory features. However, Little is known with respect to the effect and mechanism of CBD and THC and a combination of both cannabinoids on HIV infected microglia cells.

A study published by Lowe et al.^15^ proved a possible antiviral activity of CBD against Hepatitis C Virus, which was comparable to the inhibitory effect of the interferon-alpha. Similarly, 2022 Nguyen et al. suggested the inhibition of SARS-CoV-2 virus in lung cells^16^. However, a study found that CBD led to downregulating interferon-stimulated genes and decreased anti-HIV activity in recently infected macrophage cells. Interestingly, the same study reported significant reductions in HIV RNA and gag HIV protein in long-term infected cells treated with CBD^17^. In addition, DeMarino et al., reported the induction of a protective effect by reducing the release of extracellular vesicles from HIV-1 Infected monocytes and viral RNA^18^.

Agudelo et al.^19^, demonstrated that Δ(9)-THC enhanced the expression of the HIV Long Terminal Repeat gene in monocyte-derived dendritic cells isolated from human leukopacks and infected with HIV. In contrast with our results, Molina et al.^20^, reported that THC administration attenuated the progression of Simian Immunodeficiency Virus (SIV). Chronic Δ(9)-THC did not increase the viral load or exacerbate the morbidity of SIV. Moreover, Δ(9)-THC ameliorated the progression of SIV disease and attenuated the inflammation caused by the virus.

Our study did not detect an increment in GFP-positive cells, suggesting that CBD did not enhance the HIV infection; on the contrary, a reduction of GFP-positive cells and LTR gene expression was detected. However, more investigation is in need to elucidate the mechanisms behind this reduction. Specifically, our study shows that CBD significantly reduced the expression of HIV positive cells, while THC did not have this effect.

The expression of the Cannabinoid receptors (CNR) in glia cells is conditioned to the phenotype and activation of these cells. For example, CNR 1 is highly expressed in neuronal cells and lower in glial cells. On the contrary, glial cells express more CNR2. It has been reported the upregulation of the expression of CNR2 under neuroinflammation and neurodegeneration conditions in microglia cells, specifically after the stimulation with GM-CSF and IFNγ^21^ Moreover, the activation of cannabinoid receptor type 2 mitigated the neuroinflammation inducing the reduction of NLRP3^22^. In addition, HIV studies conducted in 293T cells showed the inhibition of HIV-1 LTR by synthetic, selective agonists of cannabinoid receptor 2^23^. Our results demonstrated an upregulation of CNR2 when HIV is activated and decreased the infection with cannabinoid treatments. However, there were no significant differences between the THC and CBD used alone or in combination after the induction of HIV. A different scenery was obtained when we did not treat with poly IC, and the HIV infection was not upregulated. In this case, the addition of CBD or THC did not reduce the cannabinoid receptors with respect to HIV control. However, CNR2 showed a significant dysregulation when CBD was compared against THC.

The inflammasome complex, a tool of the innate immune system, is composed of proteins responsible for activating the inflammatory process. The inflammasome activation is initiated when cytosolic pattern recognition receptors identify microbe-derived pathogen-associated or danger-associated molecular patterns generated by the host cell.

The inflammasome complexes activate effector molecules such as caspase-1 or Interleukin-1 converting enzyme, which cleaves and activates immature pro-inflammatory cytokines, interleukin IL-1β and IL-18 and Gasdermin-D^24^. The cytokines are secreted and propagate the inflammatory responses to the neighboring cells. Hence, we studied the impact of CBD compared to Δ9-THC the inflammasome activation. We specifically studied the expression of the "effector" Caspase 1, IL-1ß, and other inflammatory cytokines^14,25–27^.

Activated microglia, astrocytes, neurons, and T-cells release inflammatory mediators contributing to neuroinflammation and neurodegeneration. Furthermore, immune cells and mediators from the periphery can cross the defective BBB and augment neuroinflammation^8,9,11,28,29^. A study on HIV patients who consume cannabis, demonstrated that cannabis and its compounds decrease the number of inflammatory CD16+ monocytes, which are implicated in neuroinflammation and the secretion of pro-inflammatory cytokines and T cell activation^30–34^. Moreover, after exploring diverse functional pathways in the brains from HIV infected patients with NeuroHIV diseases, the viral loads correlated with the increase of cytokine expression^35^. Studies that reverted the latency in HIV-infected microglia cells by proinflammatory cytokines, demonstrated the activation, translocation, and binding of NF-κB p65/p50 or IRF3 to the HIV-1 promoter regions in the long terminal repeat (LTR)^29^. This may explain the reason why CBD can prevent HIV reactivation and propagation.

Neurodegeneration and neuroinflammation are caused by neurotoxic mediators and proinflammatory cytokines such as: IL-1β, IL-6, IL-8, IL-33, TNF-α, CCL2, CCL5, matrix metalloproteinase (MMPs), GM-CSF, glia maturation factor (GMF), substance P.^9,13,19,28,36,37^. Our study showed that CBD and THC led to differential effects on the inflammatory pattern at similar concentrations in HIV-infected microglial cells, with CBD producing a stronger anti-inflammatory response.

CBD significantly decreased the expression of cytokines and chemokines such as MIF, SERPIN E1, IL-6, IL-8, GM-CSF, MCP-1, CXCL1, and CXCL10 compared to THC under simillar experimental conditions. MIF, a pro-inflammatory cytokine, stimulates the production of immune mediators in the brain by microglia, astrocytes, and neurons and contributes to neuroinflammation and neurodegeneration^38–40^.

In addition, SERPIN E1, also known as Plasminogen Activator Inhibitor-1, is upregulated during inflammation, and injury. The knockdown of SERPIN E1 suppressed inflammation in a neuronal cell line from mice and TNF-α, IL-6, IL-1β, and TGF-β1 levels. Furthermore, reduced levels of SERPIN E1 were associated with inhibition of hemin-induced neuronal apoptosis^41–43^.

IL-6 is a potent proinflammatory cytokine. The upregulation of this cytokine plays a pathological impact on chronic inflammation and autoimmunity. IL-6 is recognized as a neuroinflammatory marker widely expressed in HIV infected patients^43–45^. Our study results aligned with the outcomes reported by Kozela et al. They demonstrated the decreased production of pro-inflammatory cytokines such as IL-1ß, IL-6, and IFN ß in mouse microglial cells after LPS, Δ9-THC, or CBD treatments. Furthermore, they stated that the inflammatory suppression mechanism did not involve cannabinoid receptors. Remarkably, CBD significantly reduced the activity of the NF-kB, a major regulator of the inflammation process, and the upregulated STAT3, a transcription factor implicated in inducing anti-inflammatory events. Moreover, both Δ9-THC and CBD diminished the expression of STAT1, a key regulator of IFNß -dependent proinflammatory progression^32^. Likewise, Δ9-THC reduced the expression of MCP-1 and IL-6 in the co-culture of human astrocytes and monocyte TLR-7 stimulated. These authors reported that THC acts through the CB2 receptor and suppresses IL-1ß mRNA and caspase-1 activity In the brain^46^.

MCP-1 (Monocyte chemoattractant protein-1), a beta chemokine, has been associated with various neuroinflammatory disorders, such as multiple sclerosis (MS), Alzheimer's disease (AD), and Parkinson's disease. Notably, high Serum levels of MCP-1 levels are increased in mild cognitive impairment and mild AD^47–49^. Similarly, IL-8 cytokine is associated with neuroinflammatory disorders, and CBD significantly mitigated the LPS-induced NF-κB activity, IL-8, and MCP-1 in human macrophages^50^. Our study showed similar results, Muthumalage et al. used a higher concentration of CBD. We evaluated the effect of 1uM of CBD after the activation of HIV infection with poly IC, an agonist of TLR3, in human microglia cells.

Granulocyte macrophage colony-stimulating factor (GM-CSF) generates significant activation of microglia, and causes neuronal network dysfunction, which contributes to cognitive impairments, BBB leakage, and/or cell infiltration^28,51^. GM-CSF is considered a serum inflammatory cytokine^52–55^. Moreover, in airway basal cells isolated from non-smoker HIV patients receiving antiretroviral drugs, GM-CSF is being released continuously, leading to lung inflammation^52^. Our study shows that CBD did not induce this important cytokine, unlike THC.

Additionally, we observed an upregulation in the expression of RANTES after CBD treatment (Fig. 4). CCL5/RANTES is a pro-inflammatory chemokine highly expressed in monocytes and lymphocytes, which acts as a chemotactic agent for immune cells. Previous studies concluded that RANTES has an HIV-suppressive function in a dose-dependent manner, and its antiviral function was reversed when neutralized antibodies against RANTES were used in inhibition assays^56,57^. Our study shows that CBD increases the expression of RANTES, which may attract immune cells to the infection site. This result places CBD in a preferential place for potential medical use^3,6,14,58^. On the contrary, elevated peripheral levels of CCL5 have been found in patients with major depressive disorder^59^. More studies are needed to confirm and understand the mechanisms of potential antiviral activity of CBD.

Granulocyte Colony-Stimulating Factor (G-SCF) was associated with decreased bacteremia and increased survival in neutropenic HIV-Infected patients^60^. Furthermore, it was upregulated to promote neuronal survival, proliferation, and differentiation after neuronal damage, serving as a neuroprotective mechanism^61^. Our study shows the upregulation of G-SCF.

The inflammasome process is primordial to host defense. However, it must be strictly regulated to prevent damage to the host. Therefore, it has an intrinsic regulation where a variety of pattern recognition receptors (the acidic transactivation domain, pyrin domain, caspase recruitment domain (CARD), and baculoviral inhibitory repeat (BIR)-like domains acid) can be assembled into the inflammasome complex. This regulates the production of inflammation, resulting in caspase activation as a final common pathway of inflammasome regulation. The continued activation of inflammasome has been associated with chronic inflammatory disorders such as HIV, neurocognitive disorders, etc. Therefore, reducing the triggering of the inflammasome pathway and inflammatory cytokines by CBD in the absence of psychotropic effects could be beneficial for HIV patients^8,9,11,28^. CBD has been used as an anti-inflammatory agent. However, little is known about its effect on HIV-infected glial cells. In this paper, we evidenced that CBD prevented HIV reactivation in microglia cells and inclusively reduced inflammatory cytokines such as SERPIN E1, IL-6, IL-8, and IL-1 ß under reactivation conditions. In addition, CBD deactivated the activity and gene expression of Caspase 1 at a low level of HIV infection at different time points and after 24 h of activation and treatment. On the other hand, THC showed no significant reduction in the gene expression of caspase 1 with respect to HC69.5 in the presence or absence of polyIC at 6 h, and only was significant after 24 h of added polyIC cultures. However, we could not find differences between CBD and THC in gene expression, but we did for caspase1 activity. Interestingly, the combination of both cannabinoids did not show a reduction. Moreover, the expression of NLRP3 was differentially dysregulated by these cannabinoids, whereas in infected cultures treated with CBD, a decrease of NLRP3 was detected. This reduction was not achieved in THC treated HIV infected cultures. The Caspase 1 Glo kit elicits a stable signal proportional to caspase activity, and inhibits nonspecific proteasome-mediated cleavage of the substrate. Our results demonstrated that when HIV was not reactivated, CBD and Δ(9)-THC did not enhance the caspase activity. Further, when the cells were treated with poly IC, the caspase 1 was activated, and CBD reversed that activation, not in the case of Δ(9)-THC. Thus CBD provided greater protection against caspase 1 activation, a molecule that initiates downstream cleavage to induce an inflammatory response (Fig. 5B). The regulation of inflammasome results in caspase 1 activation as a final common pathway. Rizzo and collaborators^46^ reported the downregulation of the caspase 1, IL-1 β, and NF-κB reduction in monocytes when stimulated with LPS or treated with Δ9-THC. Previously, a study reported that Δ9-THC inhibited caspase-1 activity at a concentration as low as 0.5 μM in human astrocyte-monocyte co-culture in vitro^46,62,63^. Moreover, CBD Inhibited LPS-Induced IL-1β secretion and NLRP3 Inflammasome in THP-1 Monocytes^64^. Our results support previous findings, as CBD and Δ(9)-THC did not enhance the caspase-1 activation in non-activated HIV cells, while activation of caspase 1 was detected only in HIV activated cells. Furthermore, CBD offers greater protection from the activation of the inflammasome pathway. Thus, our study corroborated with the previous reports concerning the inhibition of the IL-1β, both in latent and active HIV infected microglia cells.

There are several mechanisms proposed behind the CBD effects. One of them is the inhibition of the fatty acid amide hydrolase (FAAH), which raises the anandamide due to the absence of its breaking enzyme, FAAH. Consequently, the endogenous cannabinoid could help to reduce inflammation, pain, etc. Further, it has been shown that CBD exerts its effects by the activation of transient receptor potential cation channel subfamily V member 1 (TrpV1), which suppresses the inflammatory cytokines. Moreover, the inhibition of the NF-kB was reported due to the upregulation of its inhibitory protein IκBα. Consequently, CBD suppressed the mRNA and protein expression of NLRP3,and cytokines such as TNF-α, IFN-γ, IL-6, IL-1β, IL-2, IL-17A, and chemokines CCL-2 in T cells^14,27,58^. Corpetti et al. reported Cannabidiol as an inhibitor of the inflammasome pathway, which was activated by the stimulation of Caco-2 cells with the spike protein from SARS-CoV-2 virus. Specifically, Corpetti et al. reported that CBD reduced the expression of NLRP3 and Caspase-1^65^.

**Figure 8:**
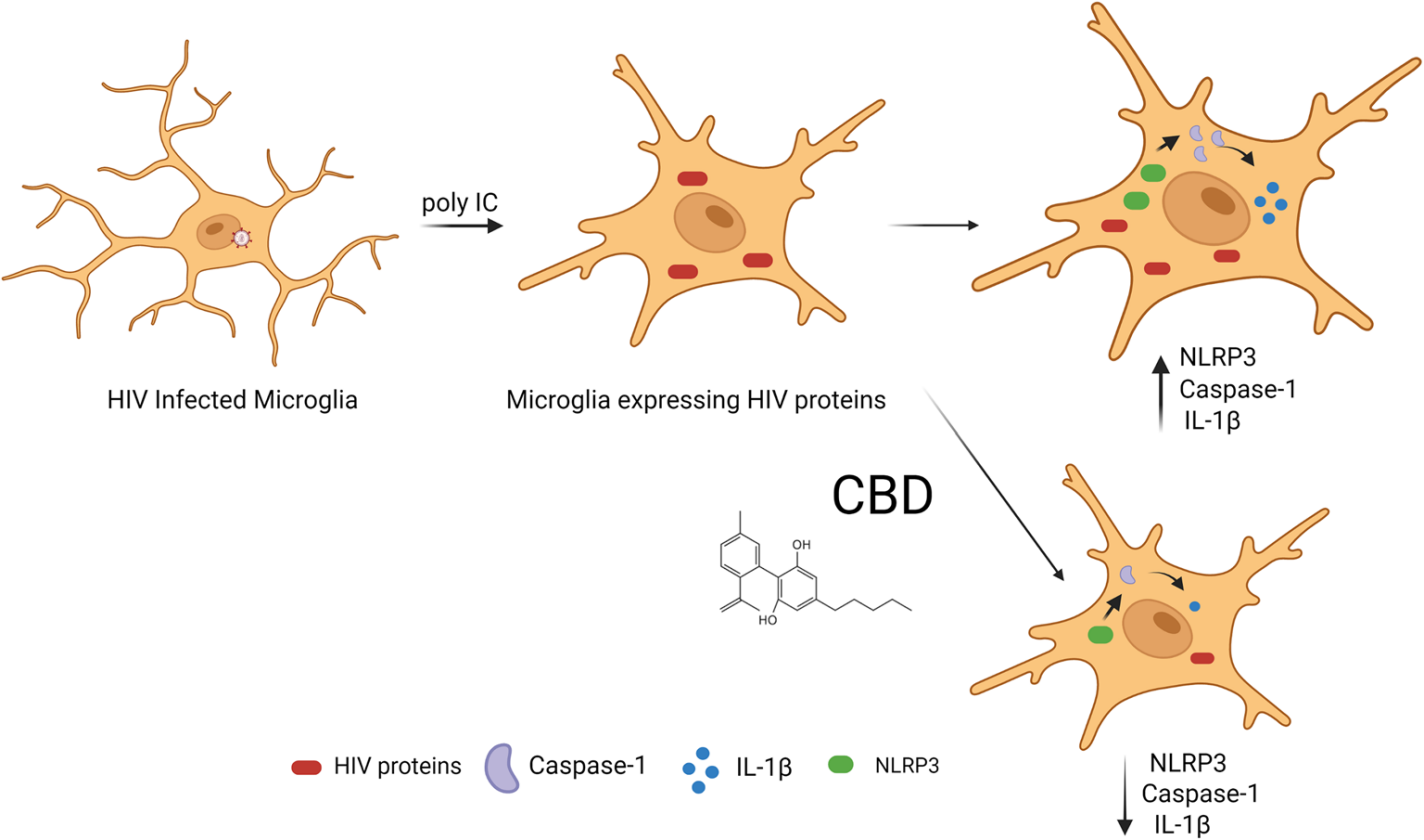
Representation of the mechanisms of action of the CBD on HIV infected microglia cells.

Additionally, in HIV-1 infection, the NLRP3 inflammasome pathway is activated and produces chronic inflammation^25–27^. A schematic representation of the mechanisms of the CBD is shown in Fig. 8.

Mboumba et al. conducted a pilot study with an oral combination of Δ9-tetrahydrocannabinol (THC): cannabidiol (CBD) (THC 2.5 mg/CBD 2.5 mg) or CBD-only (CBD 200 mg) in HIV patients under retroviral regimen. This study found that patients did not show safety concerns, and it was well-tolerated. However, there was no report about inflammatory levels, CD4 cell count, viral load, co-morbidities, etc^66^. Incorporating CBD or THC as therapeutic agents for HIV patients still requires more investigation to collect evidence on the HIV disease progression, viral latency, interaction with ART regimens, neurocognition, safety, and other co-morbidities. To date, there are no randomized controlled trials to demonstrate the potential benefits of cannabis in people with HIV.

When both cannabinoids were simultaneously used, a significant reduction of CNR2 with respect to HIV only was observed, conserving the effect of the individual drugs. Moreover, the impact on the production of caspase 1 was different. Upregulation of the expression of the gene caspase 1 and NLRP3 was observed, similar to the one found in HIV-only cultures. However, we did not detect the IL-1 β in the assayed supernatants for CBD-THC treatments after 24 h of activation and drug treatment. Al-Ghezi et al. reported an enhancement of antiinflammatory responses mediated by raised levels of IL-10 and TGF ß when a combination of CBD + THC was used in a murine model of autoimmune encephalomyelitis, multiple sclerosis. These effects were attributed to altering the gut microbiome metabolome, and anti-inflammatory cytokines were found in the serum and splenocyte supernatants^67^. Likewise, the same group of authors found that the combination of THC + CBD also showed the suppression of neuroinflammation by the dysregulation of 9 microRNAs (miR-21a-5p, miR-31-5p, miR-122-5p, miR-146a-5p, miR-150-5p, miR-155-5p, miR-27b-5p, miR-706-5p, and miR-7116) involved in anti-inflammatory response^68^. Moreover, the combination of these cannabinoids decreased the expression of TNF-α and increased Brain-derived neurotrophic factor in multiple sclerosis in vivo model^69^.

In our study, the activation of the HIV infection was induced by the adding of poly IC, leading to the activation of caspase 1 and NLRP3. Interestingly, this activation was significantly reversed by CBD, which inactivated the expression of caspase 1, a final common molecule involved in regulating the inflammasome pathway, and NLRP3. This inhibition could lead to a decrease in inflammatory cytokines such as IL-1β, IL-6, IL-8, MCP-1, SERPIN E1, etc.

In summary, our results demonstrate that CBD had a suppressive effect on the expression of LTR-HIV, CNR2, caspase 1, NLRP3, and IL-1β, after HIV activation by Poly IC. These suppressions were not observed with Δ(9)-THC, indicating a specific anti-inflammatory effect of CBD in this context. Inhibition of caspase 1, NLRP3 expression and decrease of IL-1β levels suggest a potential role for CBD in controlling the inflammasome pathway, which is involved in the regulation of the immune response. Further research is needed to fully understand the mechanisms and assess the therapeutic potential of CBD and combined cannabinoids (CBD-THC) in HIV-associated inflammation and immunological disorders.

## Conclusion

In conclusion, our study provides evidence that the beneficial outcomes of CBD have the potential as an anti-inflammatory agent in HIV-infected microglia cells. It reduces the expression of caspase-1, NLRP3, and various inflammatory cytokines such as MIF, SERPIN E1, IL-6, IL-8, and IL-1 ß in response to activated HIV infection. Further studies were needed to fully understand the mechanisms of action and the therapeutic potential of CBD in treating HIV-related neuroinflammation, eliminating the psychotropic effects of Δ9-THC. However, the results of our study suggest that CBD may be a promising candidate for the development of novel therapeutic strategies for the treatment of HIV-associated neuroinflammatory disorders.

## Material and methods

### Cell culture

HC69.5 (HIV/GFP) cells were donated by Dr. Karn’s lab. Dr. Karn’s lab team infected the cells with HIV reporter viruses. Only latently infected clones reactive to HIV in response to inflammatory signals were selected. HC69.5 (HIV/GFP) cells were rigorously characterized, and therefore provide a vital resource for the study of HIV infection in microglial cells. Cells were grown in DMEM medium supplemented with 1% FBS in a 37 °C incubator with 5% CO_2_. We selected the addition of poly IC (100 µg/ml) as an activator following previous work published by Alvarez- Carbonell^12,29^ C20, a Human microglia cell line was donated by Dr Karn’s lab and used as control^12^

### XTT cell viability assay

The XTT cell viability assay was performed to confirm that the CBD and Δ (9)-THC concentrations used were not toxic as described. Cell proliferation and viability were evaluated by the XTT assay (Cat 11465015001; Sigma Aldrich; Roche) after 24 h incubation with Δ (9)-THC and CBD (0.01 μM; 0.1 μM; 1 μM; 5 μM, 10 μM) compounds. The XTT assay was measured at 475 nm and 660 nm, using a Biotek Synergy HT multimode microplate reader instrument after the addition of XTT reagent. Results are expressed as a percentage of cell viability compared to control cells. Statistical significances were calculated by ANOVA and Dunn’s Multiple Comparison Test as a post hoc test.

### HIV reactivation

Flow cytometry analysis was performed to detect the expression of GFP as a signal for HIV activation. HIV infection in HC69.5 cells was assessed by the expression of GFP + cells. To activate the infection, we added 100 µg/ml of Poly IC following previous work published by Alvarez-Carbonell et al.^12,29^. Cells were also treated with Δ(9)-THC or CBD at two concentrations (0.01 µM and 1 µM). Cells were collected after 24 h of treatment, fixed, and permeabilized using the Fixation and Permeabilization Solution Kit (BD; Cat. No. 554715). Immortalized human microglial cells (C20, donated by Dr. Karn’s Lab)^12,29^ were used as uninfected and untreated control. Cells were acquired on an Accuri C6 flow cytometer (BD Accuri; Ann Arbor, MI; Dr. Alejandro Barbieri’s lab, FIU) and analyzed with FlowJo software (Tree Star, Inc.; Ashland, OR). Gating strategy: Forward and side scatter density plots were used to identify interested cell population and to exclude debris. Doublets were excluded by Forward scatter height vs. forward scatter area density plot. Single-parameter histograms were used to identify GFP-positive cells transferring the gate from unactivated cells. Results were expressed as numbers of positive cells.

### Quantitative real time PCR (qRT-PCR)

Gene expression was quantitated using real time qRT-PCR method. HIV infection was activated by adding 100 μg/ml of Poly IC and cells were treated with 1 μM concentration of Δ (9)-THC or/and CBD. After 24 h of treatments, total RNAs were obtained and reversed transcribed followed by real time PCR for LTR gene (HIV LTR Pa03453409_s1; Thermofisher Scientific), CNR2 gene (Hs00275635_m1), and CNR1 (Hs01038522_s1; Thermofisher Scientific). Caspase 1 (Hs00354836_m1; Thermofisher Scientific), NLRP3 (Hs00918082_m1; Thermofisher Scientific). GAPDH (Hs99999905_m1) was used as a housekeeping gene. Data represent the means ± standard error of three independent experiments. All the data were analyzed using GraphPad Prism software. The normality distribution was evaluated by Kolmogorov–Smirnov test, while statistical significance was calculated by ANOVA and Dunns Multiple Comparison Test as a post hoc test. Differences were considered significant at p ≤ 0.05.

### Proteome Profiler Human cytokine array

HC69.5 (HIV/GFP) cells were used for this study. HIV infection was activated by adding polyIC (100 μg/ml). Right after activation, cells were exposed to 1 μM of Δ(9)-THC or CBD. Supernatants from treated activated, and unactivated cells (ct = control) cells were assayed after 48 h by Proteome Profiler Human cytokine array (R&D System; Cat#ARY005B) following provider instructions. Membranes were developed, and signals were captured and recorded by ChemiDoc (Biorad). Pixel density of the dots was measured using Image J. Reuslts are presented in bar graphs for each cytokine and a picture of ech membrane. Each of the dysregulated proteins are numbered (in green) in graphs and membrane pictures. GraphPad Prism 5 software was used to graph and conduct statistical analysis. First, normality distribution was checked by the Kolmogorov–Smirnov test for each cytokines. Then, statistical significances were calculated by ANOVA followed by Dunn’s Multiple Comparison Test as post hoc tests.

### Interleukin-1 ß (IL-1 ß) ELISA

HC69.5 (HIV, GFP+) were incubated under 1% of FBS until confluence was achieved around 50%. HIV infection was activated by adding 100 μg/ml poly IC and cells were treated with 1 μM of Δ(9)-THC or CBD. Supernatants were collected after 24 h of treatment and analyzed by ELISA MAX™ Deluxe Set Human IL-1ß (Biolegend; cat# 437004).

### Caspase 1 activation

HC69.5. HIV (GFP+) cells were incubated in 1% of FBS medium until 50% confluency was achieved. HIV infection was activated by the addition of 100 μg/ml poly IC and cells were treated with 1 μM of Δ(9)-THC or CBD. After 24 h of treatment, Caspase 1 activation was analyzed using Caspase-Glo® 1 Inflammasome Assay kit (Promega; cat: G9951). Caspase-Glo® 1 Reagent or Caspase-Glo® 1 YVAD‑CHO Reagent was added to the wells, and luminescence was detected using a Biotek reader. RFUs from vehicle wells were subtracted from the signal obtained in each well. GraphPad Prism 5 software was used to graph and conduct statistical analysis. Normality distribution was checked by the Kolmogorov–Smirnov test. Then, statistical significance was calculated by ANOVA followed by Dunn’s post hoc test calculated statistical significance.

Figure 8 was elaborated using Biorender (aynda001@fiu.edu).

### Statistical analysis

GraphPad Prism software (Version 5.04; https://www.graphpad.com) was used to graph and conduct statistical analysis for all the in vitro experiments reported in this work. Normality distribution was evaluated by the Kolmogorov–Smirnov normality test. After the ANOVA was conducted Dunn's or Tukey’s Multiple Comparison Test were used as post-hoc tests. Experiments were performed at least three times in replicates.

## Data Availability

The datasets generated for this study are available on request to the corresponding author.

## Abbreviations

MIF: Macrophage Migration Inhibitory Factor
MMPs: Matrix metalloproteinase
GM-CSF: Granulocyte–macrophage colony-stimulating factor
GMF: Glia maturation factor
MCP-1: Monocyte chemoattractant protein-1
IL: Interleukin
TNF-α: Tumor necrosis Factor Alpha
IFN-γ: Interferon Gamma
Ct: Control
Poly IC: Polyinosinic–polycytidylic acid sodium salt
LTR: Long terminal repeat
CNR1: Cannabinoid receptor 1 gene
CNR2: Cannabinoid receptor 2 gene
NOD2: Nucleotide binding oligomerization domain containing 2
NLRP3: NLR family pyrin domain containing 3
